# Supplementary material for: A climate adaptation strategy for Mai Po Inner Deep Bay Ramsar site: Steppingstone to climate proofing the East Asian-Australasian Flyway
Source: PLoS One. 2020 Oct 21;15(10):e0239945. doi: 10.1371/journal.pone.0239945 (PMC7577507; doi:10.1371/journal.pone.0239945)
Supplement: Attachment — Submitted filename: Response to Reviewers.pdf [file pone.0239945.s001.pdf]

世界自然基金會
香港分會

WWF-Hong Kong

香港新界葵涌葵昌路 8 號
萬泰中心 15 樓
15/F, Manhattan Centre
8 Kwai Cheong Road
Kwai Chung, N.T., Hong Kong

電話 Tel: +852 2526 1011
傳真 Fax:+852 2845 2764
wwf@wwf.org.hk
wwf.org.hk

Ms Juliet MacAskill
PLOS ONE

22 July 2020

Dear Ms MacAskill

Thank you for coordinating this review of our manuscript, “A climate adaptation strategy for Mai Po Inner Deep Bay Ramsar Site: Steppingstone to climate proofing the East Asian-Australasian Flyway”.

We have provided the necessary attribution as requested in your last email of 20th July 2020 by adding the database in Globil under the “Creative Commons Attribution 4.0 International License”.

We have indicated this change in the letter to the handling editor (below) and highlighted this change in green.

We hope this change resolves the issue raised. Please do let us know if you require further clarifications.

Thank you again for considering this manuscript.

Sincerely

Eric Wikramanayake, PhD
Director, Wildlife and Wetlands and Lead, WWF Asian Flyways Initiative

together possible™

贊助人： 香港特別行政區行政長官
林鄭月娥女士, 大紫荊勳賢, GBS
主　席： 何闐達先生
行政總裁： 江偉智先生

義務核數師：香港立信德豪會計師事務所有限公司
義務公司秘書：嘉信秘書服務有限公司
義務司庫：匯豐銀行
註冊慈善機構

Patron: The Honourable Mrs Carrie Lam Cheng Yuet-ngor, GBM, GBS
The Chief Executive of the HKSAR
Chairman: Mr Edward M. Ho
CEO: Mr Peter Cornthwaite

Honorary Auditors: BDO Limited
Honorary Company Secretary:
McCabe Secretarial Services Limited
Honorary Treasurer: HSBC
Registered Charity
(Incorporated With Limited Liability)

註冊名稱 Registered Name: 世界自然(香港)基金會 World Wide Fund For Nature Hong Kong
(於香港註冊成立的擔保有限公司 Incorporated in Hong Kong with limited liability by guarantee)

世界自然基金會
香港分會

WWF-Hong Kong

香港新界葵涌葵昌路 8 號
萬泰中心 15 樓
15/F, Manhattan Centre
8 Kwai Cheong Road
Kwai Chung, N.T., Hong Kong

電話 Tel: +852 2526 1011
傳真 Fax:+852 2845 2764
wwf@wwf.org.hk
wwf.org.hk

Dr Yong Zhang
Academic Editor
PLOS ONE

22 July 2020

Dear Dr Zhang

Thank you for coordinating this review of our manuscript, "A climate adaptation strategy for Mai Po Inner Deep Bay Ramsar Site: Steppingstone to climate proofing the East Asian-Australasian Flyway". We also thank the reviewers for providing insightful and helpful comments to improve the manuscript.

We have attempted to address these comments as best we can, and also the changes to the acknowledgement section, statement on funding and competing interests, etc. These are provided below. (Author responses in italics).

1. **Map copyrights.** *Figure 1 has a basemap from ESRI. We have inserted the appropriate attribution in the figure caption (Basemap Sources: Esri, DeLorme, HERE) as per instructions here: https://support.esri.com/en/technical-article/000012040.*

   The landuse data in Figure 1 are prepared by WWF Hong Kong, and was also used to derive the model outputs in Figures 2 ad 3. The data are available here: https://globil-panda.opendata.arcgis.com/datasets/mai-po-habitats with a Creative Commons Attribution 4.0 International License*, and copyright is duly referenced in the manuscript.*

2. **Acknowledgements Section:** *We have removed the reference to funding and funders in the acknowledgements statement.*

   The analysis was supported by the general Mai Po Nature Reserve management fund, which is capitalized from multiple sources and managed by WWF Hong Kong.
   The salaries of EW, CO, XW, FC are supported by WWF Hong Kong (but not from the Mai Po Nature Reserve management fun).

   Authors FC and AS were provided with a consultancy by WWF HK, through Here+There Mapping Solutions to conduct the analysis.

together possible™

贊助人： 香港特別行政區行政長官
林鄭月娥女士, 大紫荊勳賢, GBS
主 席： 何闊達先生
行政總裁： 江偉智先生

義務核數師：香港立信德豪會計師事務所有限公司
義務公司秘書：嘉信秘書服務有限公司
義務司庫：匯豐銀行
註冊慈善機構

Patron: The Honourable Mrs Carrie Lam Cheng Yuet-ngor, GBM, GBS
The Chief Executive of the HKSAR
Chairman: Mr Edward M. Ho
CEO: Mr Peter Cornthwaite

Honorary Auditors: BDO Limited
Honorary Company Secretary:
McCabe Secretarial Services Limited
Honorary Treasurer: HSBC
Registered Charity
(Incorporated With Limited Liability)

註冊名稱 Registered Name: 世界自然(香港)基金會 World Wide Fund For Nature Hong Kong
(於香港註冊成立的擔保有限公司 Incorporated in Hong Kong with limited liability by guarantee)

*Please do make the appropriate changes to the online submission form based on the above information, as indicated in your instructions (i.e., "*Please include your amended statements within your cover letter; we will change the online submission form on your behalf.")

3. **Funding Statement and Competing Interests.** *Please change the Funding Statement and Competing Interests as follows:*

   "The funder (WWF Hong Kong) provided support in the form of salaries for authors [EW, CO, XW, FC], but did not have any additional role in the study design, data collection and analysis, decision to publish, or preparation of the manuscript. The specific roles of these authors are articulated in the 'author contributions' section. FC and AS received a consultancy from WWF Hong Kong through Here+There Mapping Solutions, to run the SLAMM model. This does not alter our adherence to PLOS ONE policies on sharing data and materials."
4. **Data Availability statement.** *The underlying SLAMM datalayers have been uploaded to Zenodo, and are available here:* https://doi.org/10.5281/zenodo.3935747

Reviewer's Comments to the Authors
Reviewer #1:
General comments:
1. Given the modelling nature of this manuscript, please add a paragraph or section, to discuss briefly the limitations and constrains of this SLAMM approach. Has this model been tested? What was the accuracy? A brief discussion on these will allow less-experienced readers to evaluate the evidence and follow more easily.

   Author response*: We have added some text to clarify the limitations and constraints of the SLAMM approach (lines 115-125). We also have text and provided references to show that the model has been applied to wetlands globally, including to wetlands in China and Korea, along the EAAF. We hope this will suffice.*

2. Line 352 – this is an important paragraph but this also mean it's critically important to evaluate the model's accuracy, and the probability of errors – which could lead to the purchase of less useful area. Could the authors also demonstrate how much longer will other potential buffer area (ponds around Lut Chau and Nam Sang Wai) last for? If it's just 10-20 years, then is it really a sustainable solution?

   Author response*: Presumably this refers to the paragraph starting on line 331 (in the previous, uncorrected document). Only way to show that the wetlands will survive more than 10-20 years beyond 2100 would be to run the model for another 25 and/or 50 years. But increasing the time horizon will mean that the model outputs will also become less accurate and outputs less reliable.*

   However, we also note that our recommendations to conserving Lut Chau and Nam Sang Wai fishponds do not necessarily recommend purchasing these lands, but that they not be converted to hard infrastructure now or in the near future. They are currently maintained as fishponds, which are de-facto wetlands and are used by some waterbirds as supratidal wetlands. They can also be more easily and cost-effectively restored as wetlands for

waterbirds as an adaptation strategy in the future, if not converted to hard infrastructure. Therefore, our proposal to maintain the wetlands is not on a timescale of 10-20 years beyond 2100, but starting now.

3. From the waterbirds' perspective, e.g. shorebirds, this paper could be improved substantially by considering the entire Deep Bay instead of having an arbitrary cut off in the middle of the tidal flat. Many of the birds in Maipo are also known to the mainland China side of Deep Bay and leaving the other half out could only reveal partly how waterbird's habitat will change in the future.

   Author response: *The reviewer is partly correct; the Deep Bay should be considered as an ecologically connected flyway site. However, while Mai Po and the mudflat area that was considered in the analysis is in Hong Kong SAR, the rest of the Deep Bay is under administration of the mainland China government, and we have been unable to acquire the required data layers to extend the analysis beyond Mai Po.*

   Moreover, the objective of our analysis was to identify and recommend an adaptation strategy for the Mai Po Inner Deep Bay Ramsar site. Site-scaled analyses can be more relevant, sensitive, and contextual for site-based conservation management plans. However, we then go on to recommend that similar analyses be conducted for important sites along the EAAF that can be aggregated for a holistic, flyway-scaled adaptation strategy.

   Applying the model to larger scales will result in loss of site-specific sensitivity and cause important management recommendations to be glossed over. This has been a problem with many regional and global-scaled analyses that lose site-specific context.

Specific comments:
Lines 27, 285, 393 – consider the use of "migration phenomenon" instead of "migration"
Author response: *changes made.*

Line 119 – please provide the GPS coordinate, perhaps the centroid of Maipo
Author response: *The maps (Figure 1), which is meant to be a location map, provides the coordinates.*

Line 133 – capitalize "E"ndangered
Author response: *changes made.*

Line 136 – citation needed, consider the BFS annual census report or Sung et al. 2018 www.doi.org/10.1017/S0959270917000016
Author response: *reference added*

Line 165 – please provide a bit more details since accretion can still be clearly felt today, any information on when will the land clearing and development be stopped? How much of the accretion may come from natural runoffs?
Author response: *We have tried to clarify some of the time correlation between land-clearing and development trends and accretion, as requested by the reviewer as best as possible based on available published data. However, we also note that there is no reliable literature on current accretion rates. A monitoring study is now underway, but*

the data have not been published. We have thus provided the best available information and base our choice of parameters on these studies. As such, we have used—and presented—the range of possible accretion rate scenarios in our analyses. We were unable to find any reliable information on the contributions of natural runoff-based accretion (except the historical data that we use) and that attributed to land clearing. But we also note that such a breakdown is immaterial to our analysis, except to make landuse, land conversion, and restoration recommendations, which we do in a general way. We also note that this would be an important follow up study but would have to be undertaken in collaboration with researchers from mainland China since most of the upstream impacts from runoff originates there.

Line 177 – the degree symbol looks more like a zero instead

Author response: *change made.*

Line 222 – Please explain what do low and high tides mean here (are they an average?), since substantial differences could be found between spring and neap tides.

Author response: *text added (lines change made 208-217)..*

Line 252 – may also consider the latest publication related to hunting by Gallo-Cajiao et al. 2020 https://doi.org/10.1016/j.biocon.2020.108582

Author response: *reference added*

Line 339 – may also consider the latest publication by Jackson et al. 2020 https://doi.org/10.1016/j.biocon.2020.108591

Author response: *reference added*

Reviewer #2:

As we know, the climate change and human disturbance are the main driving force of the coastal wetland change in the world. Base on the result, it seems the climate change is not the principal threat of the Mai Po till 2075. Hong Kong and Shenzhen both are the biggest city in the world. So, in the discussion, it needs to give more conservation advice or reflection, how to control or monitor the sediment. Such as, at the 2100: 2m SLR; 2mm accretion; High Tide scenario, it need build more high-tide habitat for waterbirds. Or we need to keep the sediment stabilize, try to avoid the construction and the activities of changing the ecological processes of wetland, e.g. Shenzhen Bay dredging for cruise recently.

Author response: *We agree that in addition to climate change impacts there are very severe and more proximal direct and indirect anthropogenic threats to Mai Po and the Deep Bay. Some of these can be effectively mitigated, while others would require an adaptation strategy. What we have provided through this analysis is an adaptation strategy to the expected impacts of climate change. Monitoring and tackling the more proximal anthropogenic impacts, while also critically important, is beyond the scope of this paper.*

However, we have provided general recommendations on how nature-based solutions are required in the Greater Bay in an entire sub-section entitled "Ecosystems as climate proofing solutions for Hong Kong and the Greater Bay Area" in the Discussion, especially with respect to 'climate proofing' the bay.

1. It is better put the Low tide image and Hide tide of the same scenario together and it will be easy to check the impact for the migratory waterbird.

Author response: *We debated and thought through what the best way to group the maps would be, and eventually chose to group the low tide scenarios together to show that even during low tide, important bird habitats can be submerged under low sedimentation rate scenarios. And then to show the corresponding scenarios under high tide scenarios. Thus, while we concede that there are several ways to group these maps, we have chosen what we think is the best way to show what we wanted to show.*

We hope these edits and explanations will suffice for acceptance. Please do let us know if you require further clarifications.

Thank you again for considering this manuscript.

Sincerely

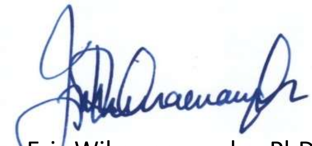

Eric Wikramanayake, PhD

Director, Wildlife and Wetlands and Lead, WWF Asian Flyways Initiative
